# Neonatal growth and breast cancer risk in adulthood

**DOI:** 10.1038/sj.bjc.6604702

**Published:** 2008-09-30

**Authors:** P Lagiou, C-C Hsieh, D Trichopoulos, H-O Adami, P Hall, L Chie, A Ekbom

**Affiliations:** 1 Department of Epidemiology, Harvard School of Public Health, 677 Huntington Avenue, Boston, MA 02115, USA; 2 Department of Hygiene, Epidemiology and Medical Statistics, School of Medicine, University of Athens, 75 M Asias Street, Athens GR-115 27, Greece; 3 Department of Medical Epidemiology and Biostatistics, Box 281, Karolinska Institutet, Stockholm SE-171 77, Sweden; 4 Division of Biostatistics and Epidemiology, UMass Cancer Center, University of Massachusetts Medical School, 364 Plantation Street, LRB 427, Worcester, MA 01605,USA; 5 Department of Obstetrics and Gynecology, Beth Israel Deaconess Medical Center, 330 Brookline Avenue, Boston, MA 02215, USA; 6 Clinical Epidemiology Unit, Department of Medicine, Karolinska Institutet, Karolinska University Hospital, Stockholm SE-171 76, Sweden

**Keywords:** breast cancer, postnatal growth, birth weight, early life, perinatal

## Abstract

Birth size of a woman has been positively associated with her breast cancer risk, particularly before menopause, but no study has investigated neonatal growth in relation to this risk. We conducted a case–control study nested within a population-based cohort of women, born in Sweden between 1901 and 1961, covering all 405 breast cancer patients and 1081 age- and hospital-matched controls, who were born after newborn charts became available. Compared to neonates who lost <200 g after birth and grew at a rate <25 g day^−1^ after reaching postnatal weight nadir (ie, the minimum, before starting to regain weight), those who either lost ⩾200 g after birth or grew ⩾25 g day^−1^ after nadir, or both, were at an approximately 50% increased breast cancer risk. The excess risk was striking and statistically significant among women below 50 years of age, but was not evident among older women. Immediate postnatal weight loss (an indicator of water loss, likely to reflect water retention associated with pregnancy hormones) as well as neonatal weight gain rate after the nadir (known to reflect growth hormone levels) was significantly positively associated with premenopausal breast cancer risk.

There is much evidence that birth size of women influences their breast cancer risk (Michels and Xue, 2006; Park *et al*, 2008), particularly before menopause (World Cancer Research Fund/American Institute for Cancer Research, 2007). No study, however, has investigated neonatal growth in relation to breast cancer risk, even though neonatal growth could be of particular importance, as it is strongly associated with neonatal IGF-1 levels (Albertsson-Wikland *et al*, 1998; Ogilvy-Stuart *et al*, 1998; Hikino *et al*, 2001; Skalkidou *et al*, 2003). IGF-1 levels, which could track through life, have been associated with breast cancer risk, particularly premenopausal breast cancer risk (Renehan *et al*, 2004; Fletcher *et al*, 2005; Rinaldi *et al*, 2006).

Evaluating neonatal growth is complicated because weight declines during the first few days after birth, mostly because of water loss, before starting to increase (Macdonald *et al*, 2003). The decline is likely to reflect the extent of water retention by the newborn at the time of delivery, under the influence of pregnancy hormones, including oestrogens (Stachenfeld and Keefe, 2002; Gomella *et al*, 2004; Stachenfeld and Taylor, 2004). The rate of weight gain after the nadir is influenced by growth factors, notably the IGF system and its determinants (Albertsson-Wikland *et al*, 1998; Ogilvy-Stuart *et al*, 1998; Hikino *et al*, 2001; Skalkidou *et al* 2003).

We have investigated neonatal growth in relation to breast cancer in adult life by a case–control study nested within a population-based cohort of Swedish women.

## Materials and methods

### Participants

In Sweden, all residents have equal access to the governmental health-care system, and because there is essentially no private in-patient treatment, hospital services are population-based. Moreover, since 1 January 1947, all residents are assigned an individually unique nine digit national registration number, which contains information on the date of birth and the county in which the individual resided in 1947 or the county of birth for those born in 1947 or later. This number allows linkage with several Swedish registries, including the Swedish National Cancer Registry (Lunde *et al*, 1980).

In the mid-1990s, we studied the intrauterine environment in relation to breast cancer risk in the offspring using information from a cohort of women who had been born in one of the five participating hospitals in the Uppsala-Örebro Health Care Region from 1874 through 1961 and who had survived at least until 1 January 1958, when the Swedish National Cancer Registry was established (Ekbom *et al*, 1997). In that study, a total of 1068 cases were diagnosed until 1994 and 2727 controls were included (Ekbom *et al*, 1997).

For this study, we retrieved newborn charts with information on postnatal growth of neonates until their discharge. The maternity wards in the five hospitals started to use newborn charts at different calendar periods, and so newborn charts were available for 406 of the 1068 eligible case patients and for 1083 of the 2727 eligible controls, all born from 1901 onwards. Because extreme prematurity has been associated with breast cancer risk (Ekbom *et al*, 2000), we excluded babies born before 32 weeks of gestation (one case and two controls), leaving 405 cases and 1081 controls. Of the former, 90 were below the age of 40 years, 168 were aged 40–49 years, 119 were aged 50–59 years, whereas 28 were aged 60–68 years. The corresponding numbers among controls were 245, 485, 290, and 61 women. In our sample, older women are underrepresented among cases because the National Cancer Registry began in 1958, when many older women belonged to cohorts born before the linked neonatal records became available. The ratio of controls to cases is lower among women 50 years of age or above at breast cancer diagnosis. Thus, among women below the age of 50 years, the control to case ratio is 2.8 (730/258), whereas among older women it is 2.4 (351/147). This is because in the earlier years, when older women were born, the likelihood of recording weight changes of newborns was much lower (when an index case was removed because of missing records, the corresponding controls were also removed, whereas if one or two controls had missing records, the remaining control(s) would suffice for retaining the corresponding case in the analysis).

At the birth of our subjects, breastfeeding predominated for newborns and the mother and child were usually discharged when the baby reached its birth weight. Generally, newborns lose weight during the first week and then gain weight (Macdonald *et al*, 2003). To examine whether these two different phases of postnatal pattern of growth were associated with subsequent risk of breast cancer, we determined maximum postnatal weight loss (defined as (birth weight)−(the lowest weight in the hospital)) and the rate of growth since the nadir (defined as (weight at discharge−weight at nadir)/(day at discharge−day at nadir)).

On the basis of the literature (Macdonald *et al*, 2003) we have created the following five mutually exclusive categories: (a) neonates who remained at the maternity wards for more than 21 days without regaining their birth weight–these neonates were analysed separately because their weight loss and gain were unusual; (b) neonates with a maximum weight loss of <200 g and growth rate after nadir <25 g day^−1^; (c) neonates with a maximum weight loss of ⩾200 g and growth rate after nadir <25 g day^−1^; (d) neonates with a maximum weight loss of <200 g and growth rate after nadir ⩾25 g day^−1^ and (e) neonates with a maximum postnatal weight loss of ⩾200 g and growth rate after nadir ⩾25 g day^−1^. All neonates in categories b–e remained at the maternity wards for a maximum of 21 days. The weight loss cutoff of 200 g was a round figure derived from the 6.6% reported to be the median percent of birth weight loss for breastfed children (Macdonald *et al*, 2003), and so with birth weight around 3000 g, we have 3000 g^*^0.066≃200 g. The cutoff for the daily rate of growth after nadir was rounded at 25 g day^−1^, as the reported median time for birth weight recovery among breastfed children is 8.3 days (Macdonald *et al*, 2003), so that 200 g divided by 8.3 days equals approximately 25 g day^−1^.

### Statistical analyses

The statistical analyses were undertaken by modelling the data through conditional logistic regression using PROC PHREG of the SAS statistical software (version 9, SAS Institute, Cary, NC, USA). Covariates adjusted in the analysis included maternal age (in years as a continuous variable), maternal socioeconomic status (low, medium, and high as an ordinal variable), maternal parity (1, 2, and ⩾3 as categorical indicator variables), pregnancy toxaemia (yes/no), neonatal jaundice (yes/no), twin membership (singleton, monozygotic, and dizygotic as categorical indicator variables), and birth weight (<2500, 2500–2999, 3000–3499, 3500–3999, and ⩾4000 g as categorical indicator variables).

The study was approved by the Institutional Review Boards of the Karolinska Institutet, Sweden, the Harvard School of Public Health, USA, and the US Department of Defense.

## Results

Table 1 presents the maternal and perinatal characteristics of breast cancer patients and their control women (matched to the cases with variable matching ratio). As reported earlier (Ekbom *et al*, 1997), neonatal jaundice is more common among cases, whereas maternal toxaemia is more common among controls. In this data set, the association between birth size and breast cancer risk was weak and statistically non-significant (Ekbom *et al*, 1997). Spearman's correlation coefficients of birth weight with maximum weight loss and daily weight gain since nadir were 0.48 (*P*<0.0001) and 0.02 (*P*=0.55), respectively. In these bivariate and possibly confounded patterns, neonatal weight loss appears more pronounced among cases than among controls. There is also some evidence that weight gain after nadir is more pronounced among breast cancer patients below the age of 50 years compared with controls.

After controlling for confounding through conditional logistic regression, we found no evidence that neonates who did not conform to the usual growth pattern are at different breast cancer risk when compared with the reference category of neonates who lost less than 200 g after birth and grew at a rate less than 25 g day^−1^ after nadir (Table 2). In contrast, however, neonates who lost ⩾200 g after birth, or neonates who grew at a rate of ⩾25 g day^−1^ after nadir, or neonates with both of these growth pattern characteristics were at an approximately 50% increased risk in later life when compared with the reference category. The excess risk was evident and statistically significant exclusively among women below the age of 50 years, who were presumably premenopausal at breast cancer diagnosis. As, in our data, women were designated as pre- or postmenopausal relying only on their age, we have evaluated whether there is an interaction between age and growth pattern with respect to breast cancer risk, and the results were of borderline significance (*P*∼0.06).

## Discussion

In our case–control study, nested within a well-defined population-based cohort of Swedish women, we have found evidence that immediate postnatal weight loss of the newborn, as well as the neonate's weight gain rate after reaching a nadir of postnatal weight, are significantly positively associated with breast cancer risk among women below the age of 50 years. As indicated in the Introduction, in the light of the literature, we considered the immediate postnatal weight loss as an indicator of water loss, probably reflecting water retention caused by pregnancy hormones, and the postnadir rate of growth as an indicator of higher levels of growth hormones, particularly IGF-1.

We interpret our findings as indicating that higher levels of pregnancy hormones and growth hormones during the immediate postnatal period, particularly IGF-1, play an important role in premenopausal breast cancer risk several decades later.

No association of postnatal growth pattern with breast cancer risk was evident among women 50 years of age or above, and presumably postmenopausal who, however, were relatively few in this study sample. Besides the numbers, it is also possible that any effect of perinatal factors on risk is gradually diluted as additional adult life breast cancer risk factors are introduced, in line with the conclusion of a major review that birth weight is positively associated with breast cancer risk mostly among premenopausal women (World Cancer Research Fund/American Institute for Cancer Research, 2007). Age at and type of menopause (natural or induced) are important postmenopausal risk factors, and pre- and postmenopausal breast cancer are frequently treated as distinct entities in studies focusing on their hormonal and non-hormonal aetiology (Hankinson *et al*, 2008).

Our study makes use of the unusual opportunities available in Sweden for linking population-based databases and registries. The nested case–control study design retains the advantages of a cohort study in terms of minimisation of information and selection bias. Exclusions were simply on the basis of the availability of linked newborn charts. The sample contained many more women below the age of 50 years (presumably premenopausal) than older women (presumably postmenopausal), and so there should be more confidence in the associations found among the former than on their absence among presumably postmenopausal women. In the base of the study, on which we relied, birth size indicators (birth weight, birth length, and placental weight) were very weakly positively related to risk, although mutual adjustment of these indicators tended to increase the positive trends (Ekbom *et al*, 1997). However, when a true but weak association is investigated in many studies, some are bound to generate non-significant or even null results (Michels and Xue, 2006; Park *et al*, 2008). We had no information about adult life risk factors for breast cancer (e.g., age at menarche), but even if associations of such factors with postnatal growth were to be found, they would probably have been placed as intermediates (which should not be controlled for) rather than as confounders (which should). There are, of course, several other risk factors (e.g., age at the first pregnancy, parity, hormone replacement therapy, and so on), which could not act as confounders, as they are unlikely to be related to postnatal growth.

It has been postulated that the likelihood of breast cancer depends on the number of mammary stem cells, which is determined in early, including intrauterine life, as well as on the early postnatal levels of growth-enhancing mammotropic hormones, which affect the replication rate of such stem cells (Trichopoulos, 1990; Adami *et al*, 1995; Trichopoulos *et al*, 2005, 2008). Birth size is known to influence breast cancer risk (Michels and Xue, 2006; Park *et al*, 2008), and there is compelling evidence that periadolescent growth (Ahlgren *et al*, 2004) and adult height (Tretli 1989; World Cancer Research Fund/American Institute for Cancer Research, 2007) are also associated with this risk. Using haematopoietic stem cells as probable correlates of the difficult-to-measure mammary stem cells, the size of their pool was positively associated with both umbilical cord growth hormones and birth weight (Savarese *et al*, 2007; Strohsnitter *et al*, 2008). No earlier investigation, however, has examined postnatal growth in relation to breast cancer risk, even though postnatal growth is rapid and the mammary gland is far from being fully differentiated (Russo and Russo, 1987).

The IGF system is associated with both breast cancer risk (Renehan *et al*, 2004; Fletcher *et al*, 2005; Rinaldi *et al*, 2006) and postnadir growth (Albertsson-Wikland *et al*, 1998; Ogilvy-Stuart *et al*, 1998; Hikino *et al*, 2001; Skalkidou *et al*, 2003), and could therefore plausibly explain the association of postnadir growth with this risk. Our explanation of the association of immediate postnatal weight reduction with breast cancer risk invokes higher levels of pregnancy hormones, including oestrogens, on the basis of well-known properties of these hormones (Stachenfeld and Keefe, 2002; Gomella *et al*, 2004; Stachenfeld and Taylor, 2004).

Replication of our results is clearly necessary. The examination of the possible differential association of neonatal growth with hormone-sensitive and hormone-insensitive breast cancer, as reflected for instance in hormone receptor expression (Duffy, 2006; Hankinson *et al*, 2008), would also be of importance. Such information was not available in our database. Animal models have provided valuable information with respect to early life exposures and breast cancer risk (Hilakivi-Clarke *et al*, 1994; Hilakivi-Clarke and de Assis, 2006) and could be useful in relation to postnatal growth.

The findings of this study are intriguing and the apparent magnitude of effect (the twofold increases in premenopausal breast cancer risk for essentially dichotomous contrasts) indicates that the phenomenon is of considerable importance. Confidence limits, however, are wide and the absence of evidence for even additive interaction is of some concern.

## Figures and Tables

**Table 1:** Maternal and perinatal characteristics of 405 breast cancer cases and 1081 matched control subjects

	**All women**				**Women <50 years old**				**Women ⩾50 years old**			
	**Cases**		**Controls**		**Cases**		**Controls**		**Cases**		**Controls**	
	***N*=405**		***N*=1081**		***N*=258**		***N*=730**		***N*=147**		***N*=351**	
	**No**	**(%)**	**No**	**(%)**	**No**	**(%)**	**No**	**(%)**	**No**	**(%)**	**No**	**(%)**
*Maternal age (years)*												
<24	125	30.9	385	35.6	77	19.0	257	23.8	48	11.9	128	11.8
25–34	213	52.6	524	48.5	138	34.1	345	31.9	75	18.5	179	16.6
35+	67	16.5	172	15.9	43	10.6	128	11.8	24	5.9	44	4.1
												
*Maternal socioeconomic status*												
Low	303	74.8	804	74.4	190	46.9	543	50.2	113	27.9	261	24.1
Medium	91	22.5	199	18.4	61	15.1	132	12.2	30	7.4	67	6.2
High	11	2.7	78	7.2	7	1.7	55	5.1	4	1.0	23	2.1
												
*Maternal parity*												
1	170	42.0	499	46.2	110	27.2	343	31.7	60	14.8	156	14.4
2	107	26.4	283	26.2	70	17.3	204	18.9	37	9.1	79	7.3
3+	128	31.6	299	27.7	78	19.3	183	16.9	50	12.3	116	10.7
												
*Maternal toxaemia*												
No	397	98.0	1036	95.8	251	62.0	703	65.0	146	36.0	333	30.8
Yes	8	2.0	45	4.2	7	1.7	27	2.5	1	0.2	18	1.7
												
*Neonatal jaundice*												
No	380	93.8	1045	96.7	248	61.2	716	66.2	132	32.6	329	30.4
Yes	25	6.2	36	3.3	10	2.5	14	1.3	15	3.7	22	2.0
												
												
*Twin membership*												
No	398	98.3	1068	98.8	253	62.5	723	66.9	145	35.8	345	31.9
Yes	7	1.7	13	1.2	5	1.2	7	0.6	2	0.5	6	0.6
												
*Birth weight (g)*												
<2500	14	3.5	42	3.9	8	2.0	26	2.4	6	1.5	16	1.5
2500–2999	57	14.1	157	14.5	36	8.9	108	10.0	21	5.2	49	4.5
3000–3499	145	35.8	408	37.7	85	21.0	279	25.8	60	14.8	129	11.9
3500–3999	143	35.3	338	31.3	101	24.9	224	20.7	42	10.4	114	10.5
⩾4000	46	11.4	136	12.6	28	6.9	93	8.6	18	4.4	43	4.0
												
*Hospital stay ⩾21 days*												
No	372	91.9	1018	94.2	244	60.2	692	64.0	128	31.6	326	30.2
Yes	33	8.1	63	5.8	14	3.5	38	3.5	19	4.7	25	2.3
												
*Maximum weight loss (g) after delivery (among normal discharge)*												
<200	73	19.6	247	24.3	48	19.7	162	23.4	25	19.5	85	26.1
⩾200	299	80.4	771	75.7	196	80.3	530	76.6	103	80.5	241	73.9
												
*Weight gain (g day* ^−*1*^ *) after reaching nadir (among normal discharge)*												
<25	183	49.2	512	50.3	119	48.8	371	53.6	64	50.0	141	43.3
⩾25	189	50.8	506	49.7	125	51.2	321	46.4	64	50.0	185	56.7
												
*Weight change after delivery (combining previous two variables)*												
<200 g/<25 g day^−1^	33	8.9	131	12.9	19	7.8	96	13.9	14	10.9	35	10.7
⩾200 g/<25 g day^−1^	150	40.3	381	37.4	100	41.0	275	39.7	50	39.1	106	32.5
<200 g/⩾25 g day^−1^	40	10.8	116	11.4	29	11.9	66	9.5	11	8.6	50	15.3
⩾200 g/⩾25 g day^−1^	149	40.1	390	38.3	96	39.3	255	36.8	53	41.4	135	41.4

**Table 2:** Conditional logistic regression-derived^a^ odds ratios (ORs) and 95% confidence intervals (CIs) for breast cancer in relation to the patterns of postnatal weight change

	**All women**			**Women <50 years old (presumably premenopausal)**			**Women ⩾50 years old (presumably postmenopausal)**		
	**OR**	**95% CI**	***P*-value**	**OR**	**95% CI**	***P*-value**	**OR**	**95% CI**	***P*-value**
*Maximum weight loss/ daily weight gain since nadir*									
<200 g/<25 g day^−1^	Reference			Reference			Reference		
⩾200 g/<25 g day^−1^	1.53	0.96–2.44	0.08	1.81	1.00–3.25	0.048	0.95	0.41–2.19	0.90
<200 g/⩾25 g day^−1^	1.39	0.78–2.45	0.26	2.33	1.13–4.78	0.02	0.50	0.18–1.39	0.19
⩾200 g/⩾25 g day^−1^	1.57	0.98–2.51	0.06	2.04	1.12–3.74	0.02	0.91	0.40–2.03	0.81
Irregulars (hospitalised for >21 days)	1.37	0.64–2.90	0.42	1.09	0.40–2.95	0.87	1.35	0.39–4.67	0.64

a Controlling for maternal age, maternal socioeconomic status, maternal parity, pregnancy toxaemia, neonatal jaundice, twin membership, and birth weight.
